# Prospective phase II trial of preoperative hypofractionated proton therapy for extremity and truncal soft tissue sarcoma: the PRONTO study rationale and design

**DOI:** 10.1186/s13014-024-02447-0

**Published:** 2024-05-14

**Authors:** Emile Gogineni, Hao Chen, Chen Hu, Karim Boudadi, Jessica Engle, Adam Levine, Curtiland Deville

**Affiliations:** 1 Department of Radiation Oncology, The Ohio State University Wexner Medical Center, 460 W 10 Ave, Columbus, OH 43210 USA; 2 Department of Radiation Oncology and Molecular Radiation Sciences, Johns Hopkins University School of Medicine, Baltimore, MD USA; 3 Department of Oncology, Johns Hopkins University School of Medicine, Baltimore, MD USA; 4 Department of Physical Medicine and Rehabilitation, Johns Hopkins University School of Medicine, Baltimore, MD USA; 5 Department of Orthopaedic Surgery, Johns Hopkins University School of Medicine, Baltimore, MD USA

**Keywords:** Sarcoma, Extremity, Preoperative, Neoadjuvant, Hypofractionated, Radiation, Proton, Particle, Protocol, Trial

## Abstract

**Background:**

Oncologic surgical resection is the standard of care for extremity and truncal soft tissue sarcoma (STS), often accompanied by the addition of pre- or postoperative radiation therapy (RT). Preoperative RT may decrease the risk of joint stiffness and fibrosis at the cost of higher rates of wound complications. Hypofractionated, preoperative RT has been shown to provide acceptable outcomes in prospective trials. Proton beam therapy (PBT) provides the means to decrease dose to surrounding organs at risk, such as the skin, bone, soft tissues, and adjacent joint(s), and has not yet been studied in patients with extremity and truncal sarcoma.

**Methods:**

Our study titled “PROspective phase II trial of preoperative hypofractionated protoN therapy for extremity and Truncal soft tissue sarcOma (PRONTO)” is a non-randomized, prospective phase II trial evaluating the safety and efficacy of preoperative, hypofractionated PBT for patients with STS of the extremity and trunk planned for surgical resection. Adult patients with Eastern Cooperative Group Performance Status ≤ 2 with resectable extremity and truncal STS will be included, with the aim to accrue 40 patients. Treatment will consist of 30 Gy radiobiological equivalent of PBT in 5 fractions delivered every other day, followed by surgical resection 2–12 weeks later. The primary outcome is rate of major wound complications as defined according to the National Cancer Institute of Canada Sarcoma2 (NCIC-SR2) Multicenter Trial. Secondary objectives include rate of late grade ≥ 2 toxicity, local recurrence-free survival and distant metastasis-free survival at 1- and 2-years, functional outcomes, quality of life, and pathologic response.

**Discussion:**

PRONTO represents the first trial evaluating the use of hypofractionated PBT for STS. We aim to prove the safety and efficacy of this approach and to compare our results to historical outcomes established by previous trials. Given the low number of proton centers and limited availability, the short course of PBT may provide the opportunity to treat patients who would otherwise be limited when treating with daily RT over several weeks. We hope that this trial will lead to increased referral patterns, offer benefits towards patient convenience and clinic workflow efficiency, and provide evidence supporting the use of PBT in this setting.

*Trial registration*: NCT05917301 (registered 23/6/2023).

## Background

Soft tissue sarcoma (STS) represents a rare and heterogeneous group of malignancies of mesenchymal origin which develops in bone and connective tissue. The American Cancer Society estimates 17,370 new cases and 7280 deaths due to cancers of the soft tissue, bone, and joints in the United States in 2023 [1]. Standard of care treatment approach for STS of the extremity and trunk typically involves limb sparing surgery and radiation therapy (RT), which has been shown to provide comparable outcomes to upfront amputation, with local control rates of 80–100% [2–4]. Moreover, multiple studies have demonstrated improved outcomes for patients with STS who are managed at high volume centers with appropriate expertise given the rarity of these tumors [5, 6].

Radiation may be delivered pre- or postoperatively using external beam radiation (EBRT), intraoperatively via a linear accelerator or brachytherapy, or using a combination of EBRT and brachytherapy. Preoperative RT may decrease the risk of joint stiffness and fibrosis after surgery due to the lower dose and smaller volumes delivered in comparison to postoperative RT while providing equivalent rates of local control [7–9]. Correspondingly, preoperative RT is now the preferred approach over postoperative RT according to the 2021 American Society for Therapeutic Radiation Oncology (ASTRO) Executive Summary [10]. This is particularly true for patients who are at higher risk for local recurrence with surgery alone, such as clinical scenarios in which there is concern for inability to achieve widely negative margins. These patients are more likely to see the benefits of decreased risk of local recurrence with radiation, with preoperative radiation associated with lower rates of irreversible late toxicities than postoperative radiation.

Wound complications continue to be a common toxicity experienced by patients who undergo preoperative RT. While modern techniques, smaller margins, and skin sparing volumes have decreased the rates of wound complications, 20–40% of patients still experience this adverse outcome, with 10–20% of patients requiring reoperation [11, 12]. Late toxicity is also a common complication which affects quality of life, occurring in 10–35% of patients treated with preoperative RT and surgical resection.

Hypofractionated preoperative RT has been shown to provide acceptable outcomes in retrospective studies and prospective trials [13–17]. Hypofractionation provides several potential advantages over conventional fractionation. These include patient convenience, shorter interval from diagnosis and treatment initiation to definitive surgical intervention, and radiobiological improvement in therapeutic ratio due to the low α/β of STS suggesting a benefit to higher doses per fraction [18, 19]. It also has the potential to increase patient referral for RT, particularly for patients who must travel long distances for daily treatment, and was shown to increase the number of patients treated with preoperative RT by three-fold at The University of California, Los Angeles (UCLA) after initiation of their trial, with a concomitant increase in the catchment area [13]. Previous prospective trials studying the use of both conventionally fractionated and hypofractionated preoperative RT in STS are summarized in Table 1.

**Table 1:** Prospective trials reporting outcomes after preoperative photon irradiation for extremity and truncal soft tissue sarcoma

Study	Year	N	Dose/fraction (frequency)	BED (Gy)*	Radiation technique	Time to surgery after RT	% of Patients receiving chemotherapy	R0 resection rate (%)	Local control	Toxicity	Wound complications
*Conventionally fractionated*											
O’Sullivan et al. [7, 8]	2002	88	50 Gy/25 fx (daily)†	75	3DCRT	3–6 wk	0	84	93%—5 yr	32%/18%/15% G2 fibrosis/joint stiffness/edema	35%, 16% requiring reoperation
Kraybill et al. [20]	2006	64	44 Gy/22 fx (daily)‡	66	Not reported	80 d	100	91	82%—3 yr	83% G4, 5% G5	11% major wound complications
O’Sullivan et al. [12]	2013	59	50 Gy/25 fx (daily)	75	IMRT	Not reported	0	93	88%—5 yr	9%/6%/11% G2 fibrosis/joint stiffness/edema	31%, 8% requiring reoperation
Wang et al. [11]	2015	79	50 Gy/25 fx (daily)§	75	75% IMRT, 25% 3DCRT	4–8 wk	0	76	94%—3.6 yr	11% late G2	37%, 0% requiring reoperation
Lansu et al. [21]	2020	79^||^	36 Gy/18 fx (daily)^¶^	54	IMRT	39–53 d (median 44)	0	94	100%—25 mo	14% late G2	22%, 17% requiring intervention
*Hypofractionated*											
Meyer et al. [22]	2014	16	28 Gy/8 fx (daily)^#^	53	Not reported	6 wk	100	94	100%—2 yr	88% G4	38% requiring reoperation
Kosela-Paterczyk et al. [15]	2014	272	25 Gy/5 fx (daily)^**^	56	3DCRT	3–7 d	22	79	81%—3 yr	15% late G2	32%, 7% requiring reoperation
Kubicek et al. [16]	2021	15^††^	35 (94%) or 40 (6%) Gy/5 fx (q2d)	96–120	Cyberknife SBRT	29–83 d (median 41)	19	80^‡‡^	93%^‡‡^—4.7 yr	27% late G1-2, 7% late G4	20% major wound complications
Gobo Silva et al. [23]	2021	18	25 Gy/5 fx (daily)	56	3DCRT, IMRT	5–18 wk (median 9)^a^	100	83	95%—29 mo	6%/6%/11% G2 fibrosis/joint stiffness/edema, 6% G3 joint stiffness, 6% G4 joint stiffness	33% major wound complications
Kalbasi et al. [13]	2022	52	30 Gy/5 fx (daily)	75	76% IMRT, 20% 3DCRT, 4% electrons	2–6 wk (median 4)	12	82^b^	94%—2 yr	16% late G2	32% requiring reoperation
Bedi et al. [24]	2022	32	35 Gy/5 fx (q2d)	96	3DCRT, IMRT	19–67 d (median 41)	31	91	100%—3 yr	22% G2 fibrosis, 13% G3 fibrosis	25% major wound complications

N, patient number; BED, biologically effective dose; RT, radiation therapy; R0 resection, surgery with negative margins; fx, fractions; 3DCRT, 3D conformal radiation therapy; wk, week(s); yr, year(s); G4, grade 4; IMRT, intensity-modulated radiation therapy; mo, month(s); G3, grade 3; G2, grade 2; d, day(s); G5, grade 5; q2d, every other day; SBRT, stereotactic body radiation therapy; R1 resection, surgery with microscopically positive margins

* Assuming α/β of 4 Gy

^†^11% of patients received postoperative boost for positive margins

^‡^Split-course irradiation; postoperative boost was given to patients with positive margins

^§^15% of patients received postoperative boost for positive margins

^||^Only included patients with myxoid liposarcoma

^¶^1% of patients received postoperative boost for positive margins

^#^6% of patients received postoperative boost for positive margins

**8% of patients received postoperative boost for positive margins

^††^16 patients were enrolled and treated with SBRT. Oncologic and toxicity outcomes reported in this table include those from the 15 patients who underwent surgical resection

^‡‡^2 of the 3 patients with initial R1 resection underwent R0 re-resection. The 1 patient with R1 resection who was unable to undergo re-resection (due to medical issues) was the only patient who experienced local recurrence in this study, which occurred in the middle of the SBRT field 100 days after surgery

^a^Patients received 3 cycles of preoperative chemotherapy every 3 weeks. Radiation commenced with the start of cycle 2. Median time between the last cycle of chemotherapy and surgery was 6 weeks

^b^5 of the 9 patients with initial R1 resection underwent R0 re-resection

**Fig. 1:**
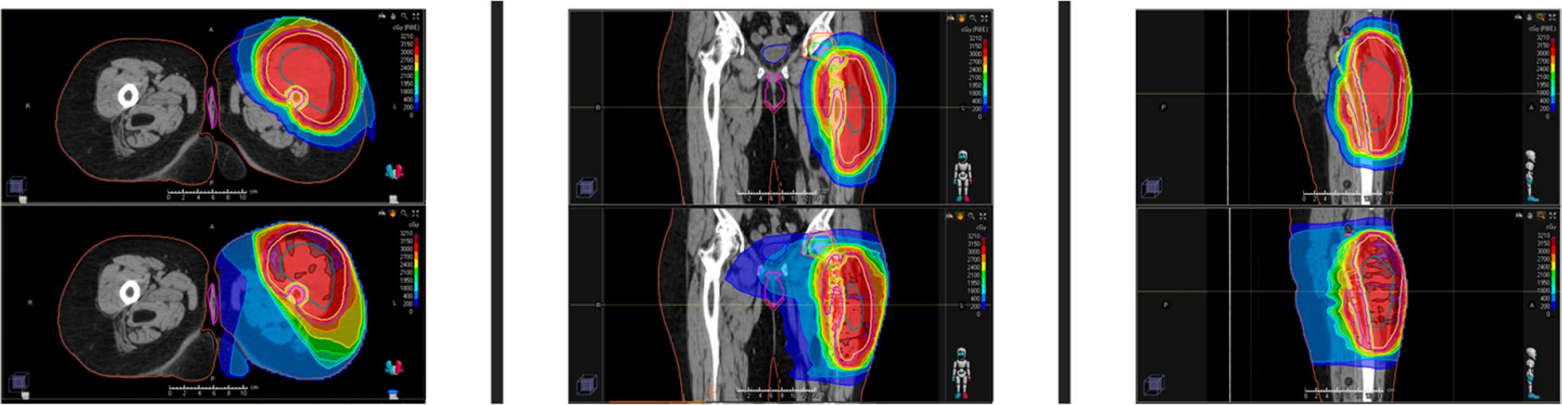
Representative color wash images in axial (left), coronal (middle), and sagittal (right) planes comparing hypofractionated preoperative proton (top row) versus photon (bottom row) dose deposition for a lower extremity sarcoma, with the dose increasing as the color ranges from blue to green to red. As shown, the target receives similar coverage in the two plans, while the normal tissue outside of the target receives lower doses in the proton plan

The traditional form of RT is with photons, which can be delivered via 3D conformal or intensity-modulated radiotherapy (IMRT) techniques. These techniques typically use multiple beams with differing angles of delivery in order to treat the target to curative doses while attempting to minimize dose to nearby organs at risk. Unfortunately, these attempts are limited by the inherent characteristics of photons, which deposit a meaningful percentage of dose beyond the target, as shown in Fig. 1.

In contrast, protons have a finite range determined by the energy with which they are accelerated, allowing the delivery of high doses of radiation with little to no dose deposited beyond the target [25]. Thus, proton beam therapy (PBT) has the potential to decrease the rates of acute and late toxicities associated with photon irradiation by limiting dose to surrounding organs at risk such as the bone, adjacent joint(s), and uninvolved fascial compartments.

One limitation of PBT is the higher entrance dose in comparison with photon irradiation. Correspondingly, this would have the potential to increase the risk of radiation dermatitis and wound complications if not accounted for. However, consideration of the proton beam orientation with the aim of minimizing exposure of tissues which will be manipulated during surgical intervention, such as incision sites and adjacent flaps, may mitigate this risk.

To explore this potential risk, our group conducted a dosimetric analysis, comparing target coverage and dose to surrounding organs at risk when treating extremity STS with identical hypofractionated regimens of photon and proton radiation as those which will be used in this trial [26]. Doses to skin were statistically similar between the two modalities for most of the dosimetric endpoints outside of mean and maximum doses, which were slightly lower with PBT than photon RT. Thus, PBT also has the potential to provide similar or potentially lower rates of wound complications and toxicity than those associated with preoperative photon irradiation.

We therefore hypothesize that employing hypofractionated PBT will further reduce RT-associated toxicity, thus serving as a highly effective method for minimizing the risk of local recurrence without incurring additional toxicity or delaying surgery for technically resectable disease.

PBT has been utilized in the treatment of STS for several decades, mostly in the setting of re-irradiation and dose escalation, however it has not yet been studied in the setting of hypofractionation [27–33]. We present a trial where patients with STS undergo preoperative, hypofractionated PBT followed by surgery. We will establish toxicity rates associated with hypofractionated PBT and explore the effects of preoperative, hypofractionated PBT on surgical complications and local recurrence-free survival (LRFS).

## Methods/design

This study titled “PROspective phase II trial of preoperative hypofractionated protoN therapy for extremity and Truncal soft tissue sarcOma (PRONTO)” is a prospective, single arm, phase II clinical trial designed to assess the safety and efficacy of preoperative, hypofractionated PBT for patients with extremity and truncal STS planned for surgical resection. We plan to accrue 40 patients who meet all inclusion and no exclusion criteria, as outlined in Table 2. The trial was activated April 2024, and we anticipate accrual to begin May 2024. With an anticipated accrual of 24–36 months for all 40 patients, we aim to complete accrual by late 2026 or early 2027.

**Table 2:** Inclusion and exclusion criteria

Inclusion criteria	Exclusion criteria
Written informed consent	History of prior local radiation therapy
≥ 18 years of age	Inability to tolerate treatment position for duration of simulation or treatment
Histologically confirmed, primary or locally recurrent extremity or truncal soft tissue sarcoma	Tumor originating in retroperitoneal location
Eastern Cooperative Group Performance Status ≤ 2	Patients planned for systemic therapy including chemotherapy, targeted agents, and/or immunotherapy
Patients planned for preoperative radiation, as determined by multidisciplinary tumor board recommendation	Co-existing malignancy or treated malignancy in the last 2 years expected to limit life expectancy; does not include completely resected cutaneous basal cell carcinoma, cutaneous squamous cell carcinoma, in situ breast or cervical malignancies, or other pathologies at the discretion of the investigators
	Confirmed pregnancy

### Trial organization

This trial was designed by the Departments of Radiation Oncology and Molecular Radiation Sciences, Oncology, Orthopaedic Surgery, and Physical Medicine and Rehabilitation of Johns Hopkins University School of Medicine. It is carried out by the Johns Hopkins Proton Therapy Center together with the Department of Radiation Oncology and Molecular Radiation Sciences at Johns Hopkins University School of Medicine. It is an investigator-initiated trial.

### Investigators

Patients will be recruited by the Departments of Radiation Oncology and Molecular Radiation Sciences and Orthopaedic Surgery of Johns Hopkins University School of Medicine. All investigators cooperating in this trial are experienced oncologists from the fields of radiation oncology and orthopaedic surgery.

### Ethical and legal considerations

The study protocol was approved by the Clinical Research Review Committee and the Institutional Review Boards of Johns Hopkins University School of Medicine (IRB00335181). The trial is carried out by adhering to local legal and regulatory requirements. All patients will sign informed consent before enrollment on trial after the nature, scope, and potential consequences of participation are explained by a physician.

### Study objectives and endpoints

The primary outcome is the rate of major wound complications occurring within 90 days after surgery, as defined according to the National Cancer Institute of Canada Sarcoma2 (NCIC-SR2) Multicenter Trial [7, 8]. This includes “secondary operation under general or regional anaesthesia for wound repair (debridement, operative drainage, and secondary wound closure including rotationplasty, free flaps, or skin grafts), or wound management without secondary operation…[including] an invasive procedure without general or regional anaesthesia (mainly aspiration of seroma), readmission for wound care such as intravenous antibiotics, or persistent deep packing for 120 days or longer.” Secondary objectives include safety and tolerability (acute grade ≥ 3 adverse events based on National Cancer Institute Common Terminology Criteria for Adverse Events version 5.0 [CTCAE v5.0]), 1- and 2-year LRFS and distant metastasis-free survival (DMFS) rates, incidence of CTCAE v5.0 late grade ≥ 2 radiation toxicity (fibrosis, lymphedema, or joint stiffness), functional outcomes using the Musculoskeletal Tumor Rating Scale (MSTS) and Toronto Extremity Salvage Score (TESS), quality of life assessed via Functional Assessment of Cancer Therapy-General (FACT-G) forms, and pathologic response (complete response, positive margins, and percentage necrosis in comparison with pre-treatment biopsy when available).

### Pretreatment evaluation

Table 3 outlines time flow for all work up, enrollment, interventions, and assessments.

**Table 3:** Time flow for all work up, enrollment, interventions, and assessments

	Screening and baseline surgical evaluation	Radiation simulation	Radiation fraction 1	Radiation fraction 2	Radiation fraction 3	Radiation fraction 4	Radiation fraction 5	Post-treatment surgical evaluation	Surgery	Post-surgery evaluation	Follow-up
*Visit windows*	Day 0	1–21 days from enrollment	7–21 days from sim	+ 2 week-days	+ 2 week-days	+ 2 week-days	+ 2 week-days	Prior to surgery	2–12 weeks from PBT completion	3–6 months post-surgery	Every 3–6 months for 2 years
Informed consent	X							X			
Medical history, demographics	X										
Inclusion/Exclusion criteria assessment	X										
*Treatment administration*											
1 fraction of 6 GyE radiation			X	X	X	X	X				
*Clinical procedures*											
ECOG performance status	X	X						X		X	X
Height and weight	X	X						X		X	X
Vital signs	X	X	X			X		X		X	X
Physical exam	X	X	X			X		X		X	X
Concomitant medications	X							X		X	X
CTCAE toxicity assessment			X			X		X		X	X
MSTS	X							X		X	X
TESS	X							X		X	X
FACT-G	X							X		X	X
Surgical resection of STS									X		
*Laboratory procedures*											
CBC w/ differential	X							X		X	X
CMP	X							X		X	X
Coagulation panel	X							X			
ESR	X							X			
CRP	X							X			
Pregnancy test*	X										
*Imaging procedures*											
CT chest or chest x-ray per SOC	X							X		X	X
CT ± MRI simulation		X									
CBCT and kV			X	X	X	X	X				
MRI and/or CT of primary site**	X	X			X		X	X		X	X

PBT—proton beam therapy, GyE—radiobiological gray equivalent, ECOG—Eastern Cooperative Oncology Group, CTCAE—common terminology criteria for adverse events, MSTS—musculoskeletal tumor rating scale, TESS—Toronto extremity salvage score, FACT-G—functional assessment of cancer therapy-general, STS—soft tissue sarcoma, CBC—complete blood count, CMP—complete metabolic panel, ESR—erythrocyte sedimentation rate, CRP—c-reactive protein, CT—computed tomography, SOC—standard of care, MRI—magnetic resonance imaging, CBCT—cone-beam CT, kV—kilovoltage X-ray

*Pregnancy test will only be done at baseline for patients of childbearing potential with uteri/ovaries

**MRI will be obtained at baseline, every 3–6 months for the first two years post-surgery, and every 6–12 months thereafter. MRI simulation is recommended when available

Initial work up includes clinical evaluation, staging computed tomography (CT) and/or magnetic resonance imaging (MRI) of the primary site, chest CT, histologic confirmation of STS, and determination of eligibility and resectability by clinical assessment and laboratory studies.

### Treatment assignment and schedule

**Fig. 2:**
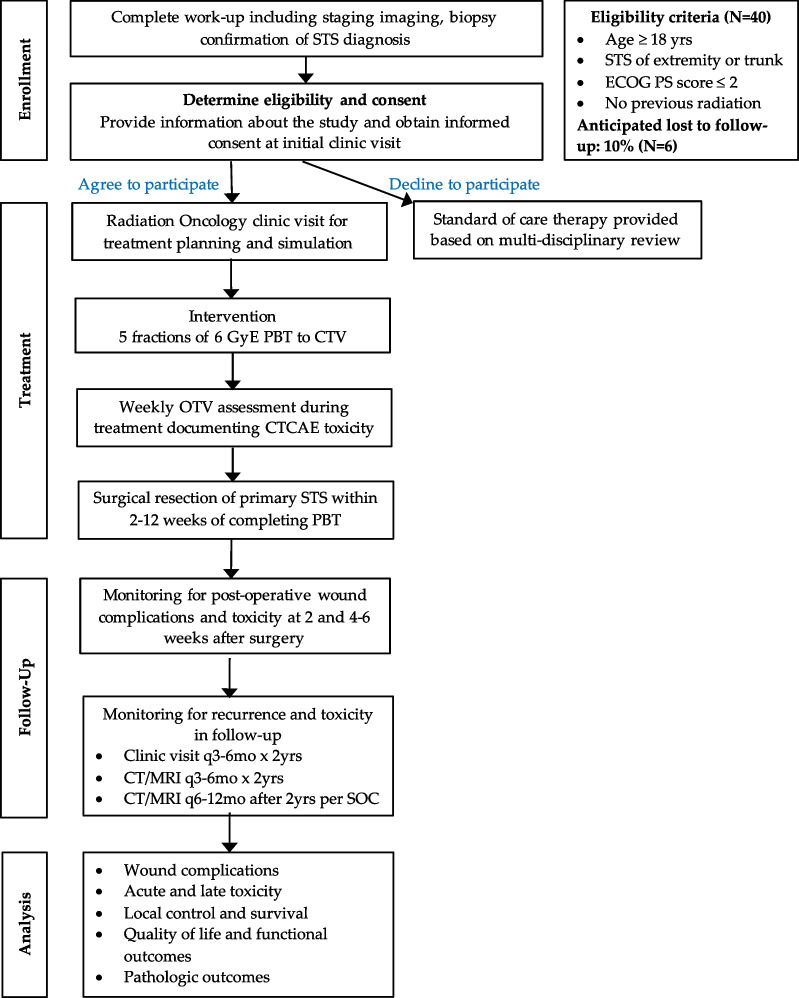
Flow chart outlining trial schema, study procedures, follow-up, and planned analysis. *Abbreviations*: STS, soft tissue sarcoma; yrs, years; ECOG PS, Eastern Cooperative Oncology Group Performance Status; GyE, Gy radiobiologic equivalent; PBT, proton beam therapy; CTV, clinical target volume; OTV, on treatment visit; CTCAE, common terminology criteria for determining adverse events version 5.0; q3-6mo x2yrs, every 3–6 months for 2 years; CT, computed tomography; MRI, magnetic resonance imaging; SOC, standard of care

All eligible patients who provide informed consent are registered and follow work-up and treatment as outlined in Fig. 2.

### Treatment details

Enrolled patients undergo CT simulation. Due to the variety of sites in which STS may occur and the mobility of extremities, careful consideration of immobilization is required prior to simulation. Immobilization must be reproducible to a high degree. Examples include the use of large vac loc or body fix bags for lower extremity immobilization with the addition of aquaplast mold to immobilize the foot and/or knee.

After completing simulation, patients begin PBT within 1–3 weeks from their simulation, treating with 5 fractions given every other weekday, to a total dose of 30 Gy radiobiological equivalent for a total of 10–12 calendar days from treatment start to completion. Clinical target volumes include gross tumor with a 3 cm margin longitudinally and 1.5 cm margin radially excluding natural barriers of spread, in addition to any surrounding edema seen on T2 weighted MRI, cropped 0.3 cm from the skin. In place of the planning target volumes (PTVs) typically used when treating with photon radiation, robust optimization of PBT is used to account for setup and range uncertainties. Target and organ at risk planning goals are listed in Table 4A, B.

**Table 4:** (A) Target dose and coverage parameters, (B) Normal tissue constraints

Target	Coverage	Total dose	Max point dose*
*(a)*			
GTV	100% of the volume to 100% of the dose	30 GyE	108%
CTV	98% of the volume to 100% of the dose	30 GyE	108%

Tissue	Constraint
*(b)*	
Normal tissue avoidance^†^	N/A
Long bones (femur, humerus)	Mean dose < 20 GyE
Femoral or humeral head	V30 GyE ≤ 5 cc
	Max dose* ≤ 33 GyE
Skin	V12 GyE ≤ 50%
2 cm longitudinal strip of skin	V12 GyE ≤ 10%
Spinal cord	Max dose* < 30 GyE
Chest wall	V30 GyE ≤ 70 cc
Bowel	V32 GyE ≤ 5 cc
Liver	V15 GyE ≤ 700 cc
Kidney	V10 GyE ≤ 10%

GTV, gross tumor volume; GyE, radiobiological gray equivalent; CTV, clinical target volume; V*x* < *y*, volume of organ at risk receiving *x* dose < *y* percentage or volume

*Maximum dose defined as dose to 0.03 cc

^†^Defined pre-treatment with input from the surgeon. The anticipated flaps or graft site to be contoured as avoidance structures as feasible [12], with the aim to arrange proton beams to minimize overlap with these structures

Patients undergo daily kilovoltage x-ray and cone beam CT (CBCT) before daily PBT to assist with treatment set-up. Quality assurance (QA) verification CT +/− MRI are performed if feasible within the first 2 fractions and repeated during the treatment course as needed.

Oncologic surgical resection occurs within 2–12 weeks of completing PBT. Patients will be followed in the postoperative setting according to standard of care surveillance for STS, as outlined in Fig. 2.

### Outcomes measured and follow-up

Medical and demographic details, performance status (Eastern Cooperative Group Performance Status scale), laboratory studies, and imaging are captured at baseline. CTCAE v5.0 toxicity, functional status using MSTS and TESS forms, and quality of life assessed by FACT-G forms are captured at baseline, during PBT, after PBT but prior to surgery, within 3 months after surgery, and every 3–6 months thereafter. Pathologic response is evaluated using rates of pathologic complete response, positive margins, and percentage necrosis in comparison with pre-treatment biopsy. Postoperative wound complications are assessed after surgery at 2 weeks, 4–6 weeks, and every 3–6 months thereafter. LRFS is assessed clinically and by postoperative MRI +/− CT of the primary site at 3 and 6 months after surgery, CT and/or MRI every 3–6 months for the first 2 years, and every 6–12 months thereafter. DMFS is assessed via chest CT or X-ray at 3 months after surgery, every 3–6 months for the first 2 years, and every 6–12 months thereafter. Patients will be followed for a minimum of 2 years on study.

### Statistical considerations

The primary outcome, rate of major wound complications, will be estimated as the number of major wound complications occurring within 90 days after surgery during the study period divided by the number of evaluable patients who have completed PBT and surgery. A sample size of 36 evaluable patients will allow us to obtain a two-sided 90% exact confidence interval with a width less than 0.3 if the incidence is between 30 and 60%, based on those reported in multiple prospective trials in patients receiving preoperative, hypofractionated photon-based EBRT followed by surgical resection for STS [13, 24].

Guarding against the potential that 10% of patients may be not evaluable, we plan to accrue 40 patients in total in order to provide 36 evaluable patients. Patients will be considered evaluable as long as they have received 1 fraction of hypofractionated PBT and completed surgery.

Safety of hypofractionated PBT in isolation will be analyzed by calculating the incidence of CTCAE v5.0 grade ≥ 3 adverse events occurring between receipt of the first fraction of PBT and the day of surgery. Specific adverse events will be reported as frequencies. Patient or cancer characteristics associated with acute PBT-related toxicity will be explored with logistic regression models provided a sufficient number of events. Tolerability will be defined and reported as the frequency of patients who stop treatment with PBT due to an adverse event.

LRFS and DMFS will be reported at 1- and 2-years post-enrollment based upon estimates produced using Kaplan–Meier methods. Patient and tumor characteristics associated with LRFS and DMFS will be explored using Cox proportional hazards models. A sensitivity analysis will be performed including patients who completed all 5 fractions of their hypofractionated PBT if this does not apply to the entire population.

### Early stopping rules

Based on existing literature, we assume the rate of major wound complication within 90 days after surgery when treated with preoperative photon RT is about 25–30%. Therefore, to minimize risks, safety will be monitored by a Bayesian stopping rule for the rate of major wound complications greater than 60%. Table 5 summarizes the continuous stopping rule for the 36 evaluable patients, evaluated in cohorts of 3 patients, starting from 6th evaluable patient.

**Table 5:** Stopping rule for safety

# Patients with major wound complication	5	6	8	10	11	13	14	16	17	19	21
# Total evaluable patients	6	9	12	15	18	21	24	27	30	33	36

At any time if the stopping criterion is met, accrual to the trial will be temporarily suspended, and the principal investigators and study team will review the toxicity data to recommend modification or termination of the trial.

## Discussion

While previous trials have provided evidence supporting hypofractionated photon RT and ongoing trials are assessing conventionally fractionated PBT, PRONTO represents the first trial evaluating the use of hypofractionated PBT for STS to our knowledge. We aim to prove the safety and efficacy of this approach and to compare our results to historical outcomes established by previous trials.

Given the low number of proton centers and limited availability, the short course of PBT outlined in this protocol is worthy of acknowledgement, as it may provide the opportunity to treat patients who would otherwise be limited when treating with daily RT over several weeks, such as those who must travel from long distances for treatment. We hope that this trial will lead to increased referral patterns, offer benefits towards patient convenience and clinic workflow efficiency, and provide evidence supporting the use of PBT in this setting.

## Data Availability

The datasets used and/or analyzed during the current study are available from the corresponding author on reasonable request.

## Abbreviations

STS: Soft tissue sarcoma
RT: Radiation therapy
PBT: Proton beam therapy
PRONTO: PROspective phase II trial of preoperative hypofractionated protoN therapy for extremity and Truncal soft tissue sarcOma
EBRT: External beam radiation
ASTRO: American Society for Therapeutic Radiation Oncology
N: Patient number
BED: Biologically effective dose
R0 resection: Surgery with negative margins
Fx: Fractions
3DCRT: 3D conformal radiation therapy
Wk: Week(s)
Yr(s): Year(s)
G4: Grade 4
IMRT: Intensity-modulated radiation therapy
Mo: Month(s)
G3: Grade 3
G2: Grade 2
D: Day(s)
G5: Grade 5
Q2d: Every other day
SBRT: Stereotactic body radiation therapy
R1 resection: Surgery with microscopically positive margins
LRFS: Local recurrence-free survival
DMFS: Distant metastasis-free survival
CTCAE v5.0: National Cancer Institute Common Terminology Criteria for Adverse Events version 5.0
MSTS: Musculoskeletal Tumor Rating Scale
TESS: Toronto Extremity Salvage Score
FACT-G: Functional Assessment of Cancer Therapy-General
CT: Computed tomography
MRI: Magnetic resonance imaging
ECOG PS: Eastern Cooperative Oncology Group Performance Status
GyE: Gy radiobiologic equivalent
CTV: Clinical target volume
OTV: On treatment visit
SOC: Standard of care
GTV: Gross tumor volume
Vx < y: Volume of organ at risk receiving x dose < y percentage or volume
CBCT: Cone beam CT
QA: Quality assurance
